# Over-Expression of IS*Aba1*-Linked Intrinsic and Exogenously Acquired OXA Type Carbapenem-Hydrolyzing-Class D-ß-Lactamase-Encoding Genes Is Key Mechanism Underlying Carbapenem Resistance in *Acinetobacter baumannii*

**DOI:** 10.3389/fmicb.2019.02809

**Published:** 2019-12-04

**Authors:** Marcus Ho-yin Wong, Bill Kwan-wai Chan, Edward Wai-chi Chan, Sheng Chen

**Affiliations:** ^1^State Key Laboratory of Chemical Biology and Drug Discovery, The Hong Kong Polytechnic University, Kowloon, Hong Kong; ^2^Department of Infectious Diseases and Public Health, Jockey Club College of Veterinary Medicine and Life Sciences, City University of Hong Kong, Kowloon, Hong Kong

**Keywords:** *Acinetobacter baumannii*, carbapenem resistance, OXA-23, OXA-51, mechanisms

## Abstract

*Acinetobacter baumannii* is an important clinical pathogen which often causes fatal infections among seriously ill patients. Treatment options for managing infections caused by this organism have become limited as a result of emergence of carbapenem resistant strains. In the current study, whole genome sequencing, gene expression studies and enzyme kinetics analyses were performed to investigate the underlying carbapenem resistance mechanisms in fourteen clinical *A. baumannii* strains isolated from two hospitals, one each in Hong Kong and Henan Province, People’s Republic of China. A large majority of the *A. baumannii* strains (11/14) were found to belong to the International Clone II (IC-II), among which six were ST208. Twelve of these strains were carbapenem resistant and found to either harbor *bla*_*OXA–*__23_/*bla*_*OXA–*__72_, or exhibit over-expression of the *bla*_*OXA–*__51_ gene upon IS*Aba1* insertion. Enzymatic assay confirmed that the OXA variants, including those of *bla*_*OXA*__–__51_, exhibited strong carbapenem-degrading activities. In terms of other intrinsic mechanisms, a weak correlation was observed between reduced production of outer membrane porin CarO/expression resistance-nodulation-division (RND) efflux AdeB and phenotypic resistance. This finding implied that over-production of carbapenem-hydrolyzing-class D-ß-lactamases (CHDLs), including the intrinsic *bla*_*OXA–*__51_ gene and the acquired *bla*_*OXA–*__23_ and *bla*_*OXA–*__24_ elements, is the key mechanism of carbapenem resistance in *A. baumannii*. This view is confirmed by testing the effect of NaCl, a known *bla*_*OXA*_ inhibitor, which was found to cause reduction in carbapenem MIC by twofolds to eightfolds, suggesting that inhibiting OXA type carbapenemases represents the most effective strategy to control phenotypic carbapenem resistance in *A. baumannii*.

## Introduction

*Acinetobacter baumannii* is an important Gram-negative pathogen that often causes serious hospital infections, especially among immunocompromised patients in intensive care units (ICUs) (Bergogne-Berezin and Towner, 1996). The increasing mortality due to *A. baumannii* infections is of major concern as this pathogen exhibits the potential to evolve into carbapenem resistant variants through acquiring antibiotic resistance-encoding mobile genetic elements, which is often exacerbated by the intrinsic low membrane permeability of this organism. These features render *A. baumannii* one of the bacterial pathogens that exhibits the highest resistance rate in clinical settings (Peleg et al., 2008). In 2013, the United States Center for Disease Control and Prevention estimated that as many as 11,500 *A. baumannii* infections occurred annually, among which 63% were multidrug resistant, resulting in 500 deaths (Queenan et al., 2012). Likewise, *A. baumannii* is responsible for more than 1/5 of all clinical Gram-negative bacterial infections in Hong Kong and other Asia-Pacific regions, with a high portion being multidrug resistant (Liu et al., 2012). Recently, the World Health Organization has listed carbapenem-resistant *A. baumannii* to be “Priority 1: Critical” in its “Global Priority List of Antibiotic-Resistant Bacteria to Guide Research, Discovery and Development of New Antibiotics,” further highlighting the worsen situation caused by this pathogen (World Health Organisation, 2017).

Carbapenem resistance in *A. baumannii* has been attributed to intrinsic cellular mechanisms, including loss of outer membrane porins (OMP) and over-expression of efflux pumps, which could result in alteration of cytoplasmic antimicrobial drug concentration and hence its bactericidal effect (Magnet et al., 2001; Siroy et al., 2005). Several OMPs, including CarO, HMP-AB and OmpW, were found to be involved in transportation of β-lactams across cytoplasmic membrane of this bacterial pathogen (Gribun et al., 2003; Siroy et al., 2006). While OMPs are responsible for the uptake of antibiotics, the multi-drug efflux systems are believed to be involved in removal of drugs by pumping them out of the cell. In particular, the resistance-nodulation-division (RND) type efflux pumps, have long been hypothesized to play a role in rendering resistance toward various antibiotics. In *A. baumannii*, the most extensively studied RND efflux system is the *adeABC* gene product, which exhibits substrate specificity toward various β-lactams, including fluoroquinolones, aminoglycosides, tetracyclines and chloramphenicol (Higgins et al., 2004). Nevertheless, evidence confirming a direct linkage between carbapenem susceptibility and the presence/absence of these porin proteins and efflux systems in *A. baumannii* is currently not available.

Enzymatic mechanisms have been regarded as the key factors that mediate development of carbapenem resistance in Gram negative bacteria, including *A. baumannii*. Instead of *bla*_*IMP*_, *bla*_*VIM*_ and *bla*_*NDM*_ which are commonly identified in other bacterial pathogens, the carbapenem-hydrolyzing-class-D β-lactamases (CHDLs) are regarded as key determinants underlying the emergence of carbapenem-resistant *A. baumannii* (Poirel and Nordmann, 2006). CHDLs denote the OXA-type β-lactamases which exhibit carbapenem hydrolyzing activity. There are various types of *bla*_*OXA*_ genes which are known to be harbored by *A. baumannii*, including *bla*_*OXA–*__51_, *bla*_*OXA–*__23_, *bla*_*OXA–*__24__/__40_, *bla*_*OXA–*__58_, *bla*_*OXA–*__143_, and *bla*_*OXA–*__235_ (Higgins et al., 2013; Evans and Amyes, 2014). A considerable number of studies have been conducted on enzymes encoded by these resistance genes due to their uniqueness in *A. baumannii*, particularly the *bla*_*OXA–*__51_-like β-lactamases, the genetic determinant of which is inherent in *A. baumannii* chromosome and can be readily overexpressed as a result of promoter activation by insertion sequences such as IS*Aba1* (Turton et al., 2006). Apart from this chromosomal resistance gene, plasmid-borne *bla*_*OXA–*__23_-like, *bla*_*OXA–*__24__/__40_-like, and *bla*_*OXA–*__58_-like elements are also frequently identified in resistant isolates. Among them, *bla*_*OXA–*__23_ is the most prevalent CHDL-encoding element in carbapenem-resistant *A. baumannii* worldwide (Mugnier et al., 2010). A previous study in China reported that 96.5% of carbapenem-resistant *A. baumannii* isolates carried *bla*_*OXA–*__23_-like elements, and that over 96% of those isolates belonged to the Clonal Complex CC92 (Ji et al., 2014). In Hong Kong, the majority of carbapenem-resistant *A. baumannii* were *bla*_*OXA–*__23_-like carrier that belonged to sequence type ST26 (Ho et al., 2010). Similarly, dissemination of *bla*_*OXA–*__23_-bearing *A. baumannii* was also observed in other Asian countries, including Taiwan, Japan, and Korea (Peleg et al., 2008).

A comprehensive study was performed in 2013 to investigate the interplay between intrinsic and extrinsic mechanisms in mediating development of antimicrobial resistance in *A. baumannii*, including efflux systems, membrane porins, and production of CHDL β-lactamases (Rumbo et al., 2013). The study concluded that the *bla*_*OXA–*__24_ and *bla*_*OXA–*__58_ are the major determinants of carbapenem resistance in this organism, and that *bla*_*OXA–*__51_ and porin proteins were not involved in antimicrobial resistance of *bla*_*OXA–*__24_ and *bla*_*OXA–*__58_-carrying isolates (Rumbo et al., 2013). Nevertheless, the exact role of intrinsic mechanisms in mediating carbapenem resistant phenotypes in *A. baumannii* strains carrying *bla*_*OXA–*__23_ and *bla*_*OXA–*__51_ remained unclear. In this work, we showed that intrinsic resistance mechanisms including RND-mediated efflux and reduced expression of porin proteins did not play a major role in mediating onset of carbapenem resistance in *A. baumannii.* Instead, the phenotype was mainly conferred by CHDL encoded by the *bla*_*OXA–*__23_, *bla*_*OXA–*__72_, and *bla*_*OXA–*__51_-like genes. In addition, we identified several variants of *bla*_*OXA–*__51_, which exhibited carbapenem-hydrolyzing activities that resemble those encoded by *bla*_*OXA–*__23_ in terms of substrate profile, as over-expression of these *bla*_*OXA–*__51_ variants in *A. baumannii* upon insertional activation by IS*Aba1* conferred the host strain a carbapenem resistant phenotype identical to *bla*_*OXA–*__23_-carrying strains.

## Materials and Methods

### Bacterial Isolates

A total of 14 representative *Acinetobacter baumannii* clinical strains were first included in the genome sequencing, gene expression study, and western blot analysis as described below. The strains were isolated from patients of two hospitals, one each in Hong Kong and Henan Province, People’s Republic of China, during the period between 2000 and 2013. These strains exhibited various carbapenem resistance phenotypes and genotypes. The genetic identity of these isolates was confirmed by the Vitek II bacterial identification system prior to further analysis. The ethic approval for this study was covered by human subject ethic approval, 2018-039, approved by the Second Affiliated Hospital of Zhejiang University, Zhejiang, China. An addition 453 clinical carbapenem-resistant *A. baumannii* strains isolated from four different regions of China, were included in the screening of *bla*_*OXA*_ genes in the latter part of the study. The experimental flow is illustrated by Supplementary Figure S1.

### Antimicrobial Susceptibility Test

Minimal inhibitory concentration (MIC) of carbapenems was tested for the 14 *Acinetobacter baumannii* strains by using the microdilution method and interpreted according to CLSI guidelines (Clinical and Laboratory Standards Institute, 2016). Briefly, bacterial strains were grown on MH agar. Bacterial cell suspensions at a concentration equivalent to a 0.5 McFarland Standard were prepared and inoculated into microplate wells containing different concentrations of carbapenem. The final volume in each well was 150 μL. The effect of the efflux pump inhibitor Phenylalanine-arginine β-naphthylamide (PAβN) and CHDL inhibitor Sodium chloride on the susceptibility of the test strains to carbapenem was determined by adding the compounds to specific wells of the microtiter plate to produce a concentration of 30 μg/ml and 200 mM, respectively. The MIC test was repeated twice to ensure the accuracy of the result.

### Whole Genome Sequencing and ST Typing of *Acinetobacter baumannii*

Whole genomic DNA was extracted from the strains using the Invitrogen^TM^ PureLink^TM^ Genomic DNA Mini kit, followed by sequencing with an Illumina^®^ NextSeq 550 system. Raw reads generated in this study and the Illumina reads of 11 strains obtained from the NCBI database were trimmed or filtered to remove low-quality sequences and adaptors. Genome data were annotated with the RAST tool (Overbeek et al., 2014) and Prokka (Seemann, 2014). Scaffolds obtained were analyzed by Geneious 9.7. *A. baumannii* type strain ATCC19606 was used as reference throughout the analysis. ST profiles were determined according to the *A. baumannii* MLST (Oxford) database using sequences extracted from whole genome sequencing. Discrimination of International Clone II was based on analysis of the *bla*_*OXA–*__51_ gene as described previously (Matsui et al., 2013).

### qRT-PCR Analysis on Gene Expression

Overnight culture of *A. baumannii* isolate was inoculated into fresh LB broth until OD 0.6 was reached. Total RNA was extracted using the QIAGEN RNeasy^®^ Mini Kit. The extracted RNA was further treated with the Invitrogen Turbo DNA-free^TM^ Kit to remove any DNA contaminants. 1 μg of purified mRNA samples were reverse-transcribed to cDNA by using the Invitrogen^TM^ SuperScript^TM^ III First-Strand Synthesis SuperMix kit for qRT-PCR. Real-time PCR was performed on a Roche LightCycler^®^ 480 System and *Power*SYBR^TM^ Green PCR Master Mix was used as the reaction medium. The parameters of PCR were as followed: the reaction mixture was first incubated at 95°C for 10 min for complete denaturation of template and activation of DNA polymerase; followed by 40 cycles of denaturation at 95°C for 10 s, annealing and polymerization at 60°C for 1 min. The melting temperature of PCR product was measured after completion of PCR to ensure the absent of non-specific PCR product. Expression level of endogenous genes was normalized with *gyrB* and *A. baumannii* ATCC19606 was used as control for comparison. The gene expression study was performed in triplicate. Primers used in this study are listed in Supplementary Table S1.

### Western Blot of *bla*_*OXA–*__51_-Like Proteins

Cell lysates were solubilized by boiling with SDS running buffer for 10 min and subsequently separated by SDS-PAGE with 12% separating gel. Proteins were transferred to a PVDF membrane followed by blocking by skimmed milk for 1 h and incubated with mouse anti-OXA-51 antibody at 4°C overnight. The goat anti-mouse antibody was used as the secondary antibody. The signal was generated by HRP substrate and detected by chemiluminator. The broad range anti-GADPH (ABCAM) was used as loading control.

### Further Screening of Carbapenem-Resistant *Acinetobacter baumannii* Clinical Isolates

An addition 453 clinical carbapenem-resistant *A. baumannii* strains, isolated from four different regions of China, were further screened for the presence of *bla*_*OXA–*__23_, presence of IS*Aba1* in the promoter region of *bla*_*OXA–*__23_, *bla*_*OXA–*__51_ and presence of IS*Aba1* in the promoter region of *bla*_*OXA–*__51_. The primers sequences are shown in Supplementary Table S1. To screen for the presence of IS*Aba1* in the promoter region of *bla*_*OXA–*__23_ or *bla*_*OXA–*__51_, PCR assay was performed using primer IS*Aba1*-F was used together with reverse primer of *bla*_*OXA–*__23_ or *bla*_*OXA–*__51_. Strains that did not contain *bla*_*OXA–*__23_ with insertion of IS*Aba1* in the promoter region were selected for Western-blot analysis as mentioned above to check for the over-expression of OXA-51 in these strains.

## Results and Discussion

### Antimicrobial Susceptibility and Sequence Analysis of *Acinetobacter baumannii* Strains

Fourteen clinical *A. baumannii* strains were first tested for their susceptibility toward carbapenems. Resistance toward imipenem and meropenem was observable in 12 of the 14 strains, with MIC ranging from 8 to ≥64 μg/ml (Table 1). The fourteen strains were then subjected to whole-genome sequencing. Based on the sequences obtained, about half of the strains were found to belong to ST208 (6 out of 14), whereas three strains (AB2, AB5, and MH5) belonged to ST940. For the remaining five strains they belonged to different ST types. Only the three ST940 strains were found to belong to International Clone II (IC II) based on analysis on the intrinsic *bla*_*OXA–*__51_ gene harbored by these strains (Table 1 and Supplementary Figure S6). The findings corroborated with those in previous studies which reported the dissemination of *A. baumannii* Clonal Complex 92 strains within the Asia Pacific region, including Taiwan, South Korea and China (Kim et al., 2013). It has previously been demonstrated that the high genome plasticity of *A. baumannii* may result in sporadic loss of endogenous genes, altering the physiological status and hence antibiotic susceptibilities of the organism (Roca et al., 2012). In view of the possibility that resistance formation is due to loss of specific physiological functions, the relationship between presence of various intrinsic determinants of carbapenem sensitivity was determined; the determinants tested included the outer membrane porin-encoding genes *carO* and *ompA*, as well as the efflux gene *adeABC* and its regulator *adeRS*. It was found that all the test strains carried the intact *carO* and *ompA* genes without insertion of IS elements; this finding was consistent with that of a previous report (Rumbo et al., 2013). However, three strains (AB1, AB3, and MH5) were found to lack *adeRS*, with the latter two also lacking *adeB*. The intrinsic *bla*_*OXA–*__51_ gene was detectable in all test strains, among them, the three ST940 strains were found to carry the variant gene *bla*_*OXA–*__99_, whereas the *bla*_*OXA–*__66_, *bla*_*OXA–*__79_, *bla*_*OXA–*__82_ and *bla*_*OXA–*__83_ variants were identified in the other strains. Interestingly, the IS*Aba1* element was found to be inserted into the promoter region of *bla*_*OXA–*__51_ in five strains, all of which belonged to IC-II. Apart from carriage of various intrinsic CHDL genes, half of the strains were found to have acquired the *bla*_*OXA–*__23_ gene, two of which were located in the chromosome. One strain was also found to harbor the *bla*_*OXA–*__72_ element (a variant of *bla*_*OXA–*__24_) (Table 1). It has been described that alteration of amino acids of the *adeRS* gene product could result in change of expression level of *adeB* (Wen et al., 2017). Sequences of *carO*, *adeB* and *adeRS* were then analyzed and aligned in attempt to establish their relationship with carbapenem susceptibility. However, it was shown that the genetic differences detectable in these genes were linked closely to their genetic types (IC-II and non IC-II) rather than carbapenem sensitivity (Supplementary Figures S2–S5). Other known carbapenemase genes such as *bla*_*NDM–*__1_ were not detectable in the test strains. The combinational analysis of carbapenem resistance genotypes and their genetic characteristics showed that strains exhibiting carbapenem resistance were either carrying an additional CHDL gene (*bla*_*OXA–*__23_ and *bla*_*OXA–*__24_) or having an IS*Aba1* linked upstream region in their *bla*_*OXA–*__51_ element. Consistently, two carbapenem-susceptible isolates carried neither *bla*_*OXA–*__23_ or *bla*_*OXA–*__24_ nor insertion of IS*Aba1* in the intrinsic *bla*_*OXA–*__51_ (Tables 1, 2).

**TABLE 1:** Genetic and phenotypic characteristics of 14 *Acinetobacter baumannii* clinical strains tested in this study.

**Strain**	**Year/Origin**	**ST type (Oxford)**	**International clone**	**Presence of resistance determinants**							**MIC (μg/ml)**				
					
				***carO***	***adeB***	***adeRS***	***bla*_*OXA–*__51_ variants**	**IS*Aba1*-*bla*_*OXA–*__51_**	***bla*_*OXA*−__23__/__24__/__40_**	**IS*Aba1*-*bla*_*OXA*__23_**	**IMP^@^**	**IMP/PAßN**	**MER^@^**	**MER/PAßN**	**COL**
AB1	2004, HK	208	IC II	+	+	−	OXA-83	+	−	−	16 (4)	16	32 (8)	32	1
AB2	2008, HK	940	Non-IC II	+	+	+	OXA-99	−	OXA-23	+	16 (8)	16	32 (16)	16	2
AB3	2011, HK	208	IC II	+	−	−	OXA-66	+	OXA-23^#^	+	32 (8)	32	≥64 (32)	32	2
AB4	2011, HK	253	IC II	+	+	+	OXA-66	−	OXA-23	+	≥64 (8)	≥64	≥64 (32)	≥64	2
AB5	2004, HK	940	Non-IC II	+	+	+	OXA-99	−	OXA-23	+	16 (4)	16	8 (8)	8	1
AB7	2011, HK	208	IC II	+	+	+	OXA-82	+	−	−	8 (4)	8	16 (16)	16	8
AB8	2003, HK	208	IC II	+	+	+	OXA-82	+	−	−	8 (8)	8	32 (16)	16	2
AB10	2000, HK	208	IC II	+	+	+	OXA-66	−	−	−	1 (0.25)	0.5	0.25 (0.25)	0.5	2
MH1	2000, HK	684	IC II	+	+	+	OXA-66	−	−	−	0.5 (0.5)	1	0.25 (0.125)	0.5	0.5
MH2	2010, HK	218	IC II	+	+	+	OXA-66	−	OXA-72	−	≥64 (64)	≥64	≥64 (≥64)	64	1
MH3	2011, HK	208	IC II	+	+	+	OXA-79	+	−	−	8 (4)	8	32 (8)	16	2
MH5	2006, HK	940	Non-IC II	+	−	−	OXA-99	−	OXA-23	+	32 (16)	16	32 (8)	4	1
MH6	2013, HN	195	IC II	+	+	+	OXA-66	−	OXA-23	+	32 (16)	16	32 (8)	8	2
MH7	2013, HN	369	IC II	+	+	+	OXA-66	−	OXA-23^#^	+	≥64 (32)	≥64	≥64 (16)	32	2

*Presence of endogenous genes and other resistance determinants were investigated by analysis of whole genome sequences.^@^MIC performed in the presence of NaCl.^#^Chromosome-based. HK, Hong Kong; HN, Henan province, China. IMP, imipenem; MER, meropenem; COL, colistin; PAβN, Phenylalanine-arginine β-naphthylamide.*

**TABLE 2:** Expression level of genes related to carbapenem resistance in *A. baumannii* strains.

**Strain**	**Relative expression level of different genes by qPCR**				
	
	***carO***	***adeB***	***adeR***	***bla*_*OXA–*__51_**	***bla*_*OXA–*__23_**
ATCC19606	1	1	1	1	n.a
AB1	1.53 ± 0.10	0.07 ± 0.01	n.a	25.75 ± 2.85	n.a
AB2	0.79 ± 0.09	0 ± 0.00	0.91 ± 0.01	1.53 ± 0.21	3.93 ± 0.31
AB3	1.1 ± 0.05	n.a	n.a	25.09 ± 3.06	3.14 ± 0.11
AB4	0.54 ± 0.06	0.04 ± 0.00	1.37 ± 0.02	0.98 ± 0.32	2.24 ± 0.06
AB5	0.53 ± 0.06	0 ± 0.01	0.92 ± 0.01	1.8 ± 0.10	3.82 ± 0.39
AB7	2.86 ± 0.12	0.31 ± 0.01	2.74 ± 0.21	58.26 ± 4.87	n.a
AB8	2.29 ± 0.11	0.25 ± 0.02	2.16 ± 0.16	53.27 ± 4.03	n.a
AB10	0.74 ± 0.01	0.2 ± 0.02	3.63 ± 0.28	2.81 ± 0.26	n.a
MH1	0.27 ± 0.01	0.12 ± 0.01	1.04 ± 0.08	2.46 ± 0.57	n.a
MH2	1.39 ± 0.15	0.2 ± 0.02	2.84 ± 0.30	1.39 ± 0.13	n.a
MH3	2.33 ± 0.15	0.17 ± 0.01	2.89 ± 0.19	64.96 ± 5.07	n.a
MH5	1.18 ± 0.28	n.a	n.a	7.16 ± 0.65	4.37 ± 0.36
MH6	1.13 ± 0.13	0.15 ± 0.01	3.44 ± 0.36	2.99 ± 0.45	7.64 ± 0.72
MH7	1.43 ± 0.13	0.13 ± 0.01	3.27 ± 0.50	2.25 ± 0.22	5.10 ± 0.16

*Expression levels were normalized with ATCC19606 for *carO*, *adeB*, *adeR* and *bla*_*OXA–*__51_. Strains lacking the corresponding genes were depicted by “n.a.” Expression of *adeB* was not detected in AB2 and AB5.*

### Efflux Pumps Did Not Mediate Carbapenem Resistance in *Acinetobacter baumannii*

The effect of RND-efflux systems on carbapenem susceptibility in *A. baumannii* clinical strains was evaluated by supplementing Phenylalanine-arginine β-naphthylamide (PAβN) at a concentration of 30 μg/ml in determination of MIC toward carbapenem. As shown in Table 1, we found that addition of PAβN exhibited minimal effect on altering carbapenem MIC except for two strains (Table 1). This finding demonstrated that RND efflux pumps only play a minimal role in mediating carbapenem resistance in *A. baumannii* strains of hospital origins and that the resistance phenotype is mainly conferred by the *bla*_*OXA*_ enzymes.

To further investigate the role of various host determinants in mediating changes in carbapenem susceptibility, the expression level of various putative resistance genes, including the RND efflux pump gene *adeB*, its regulator *adeR*, the outer membrane porin gene *carO*, as well as the *bla*_*OXA–*__23_ and *bla*_*OXA–*__51_-like β-lactamase-encoding elements, were further analyzed by RT-qPCR. The expression level of each gene was normalized by the gene encoding the gyrase protein subunit B (*gyrB*) of the *A. baumannii* type strain ATCC19606. The results are shown in Table 2 and Supplementary Figures S7–S11. For *carO*, varied expression level was observed among the test strains, with most isolates exhibiting a level similar to or lower than that of ATCC19606. For the two susceptible strains, AB10 and MH1, *carO* expression was lower than that of ATCC19606 and most of the other carbapenem-resistant counterparts. The result is in line with a previous study, in which a decreased expression of *carO* has been described in carbapenem susceptible isolates recovered from Brazil (Fonseca et al., 2013). It should be noted that although strains AB7, AB8, and MH3 exhibited elevated *carO* expression by an amplitude >twofolds, their contribution to carbapenem resistance was hard to be addressed due to the presence of other major resistance mechanism, presence of OXA types of carbapenemases (Tables 1, 2).

An unexpected phenomenon regarding the *adeB* expression level of the test strains was observed. It was proposed that elevated expression of *adeABC* might contribute to reduced carbapenem susceptibility, possibly due to enhanced extrusion of intracellular antibiotics through efflux activity (Kim et al., 2013). Nevertheless, except for the two test strains AB3 and MH5, which lacked *adeB*, all other test strains exhibited a very low transcript level of this efflux gene, with a range of 0.07 to 0.31 compared to ATCC19606. Transcript of *adeB* was not detected in strains AB2 and AB5. Of note, *adeB* expression in the two carbapenem sensitive strains AB10 and MH1 was similar to most of their resistant counterparts. In order to evaluate whether decreased *adeABC* expression was due to effect of transcription regulation, expression level of *adeR* was also examined in an attempt to determine the degree of correlation between the two. Contrary to the data on *adeB*, similar or higher transcription level of *adeR* was observed among the majority of the test isolates, ranging from 0.91 to 3.63 folds when compared to ATCC19606 (Table 2). Correlation between expression of *adeB* and *adeR*, as well as *adeR* and carbapenem susceptibility, therefore could not be established. Taken together, the results suggest that the *adeABC* gene plays a negligible role in mediating changes in carbapenem susceptibility, particularly in strains which harbor additional CHDL genes such as *bla*_*OXA–*__23_ and *bla*_*OXA–*__24_. Indeed, the results of genotypic analysis corroborated with those of the expression study in that a lack of *adeABC* efflux system was observed in several resistant strains.

### Overexpression of OXA-23 and Intrinsic OXA-51 Through IS*Aba1* Insertion Was the Major Mechanisms of Carbapenem Resistance

**FIGURE 1:**
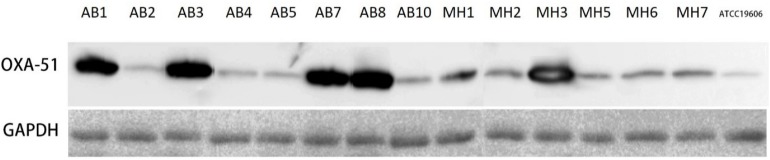
Western blot analysis of *Acinetobacter baumannii* strains using OXA-51 specific antibody. The broad-range anti-GAPDH was used as loading control.

Antimicrobial susceptibility test revealed that all *bla*_*OXA–*__23_ carriers were resistant to imipenem and meropenem but exhibited varied MIC values, presumably due to the fact that these strains carried various variants of *bla*_*OXA–*__23_ and *bla*_*OXA–*__51_. Expression level of *bla*_*OXA–*__51_ and *bla*_*OXA–*__23_ was examined in an attempt to determine the degree of contribution of carbapenemases encoded by these genes to reduction in carbapenem susceptibility of the *A. baumannii* strains tested in this study. All *bla*_*OXA–*__23_ genes were found to carry insertion in the promoter region by IS*Aba1*. Consistently, expression level of the *bla*_*OXA–*__23_ gene was found to be up-regulated 2.24 to 7.64 folds among the strains. Discrepancies between the effects of plasmid-borne and chromosome-based elements were not observed (Tables 1, 2). Among the 6 *bla*_*OXA–*__23_ carriers which lacked an over-expressed *bla*_*OXA–*__51_, MIC values of imipenem and meropenem ranged from 16 to ≥64 μg/ml and 8 to ≥64 μg/ml, respectively. In particular, the higher transcript level of *bla*_*OXA–*__23_ did not necessarily confer higher resistance level toward carbapenems. Since identical *bla*_*OXA–*__23_ was harbored by all the strains, the possibility of varied activity toward carbapenem in individual variants can be ruled out. The lack of association between *bla*_*OXA–*__23_ expression and susceptibility might then be attributed to physiological status of individual *A. baumannii* strains. Result of *bla*_*OXA–*__51_ expression revealed consistency between IS*Aba1* insertion and transcript level of this endogenous carbapenemase. For the five test strains which carried the IS element, *bla*_*OXA–*__51_ expression ranged from 25.09 to 64.96 folds compared to ATCC19606. To ensure that the overexpression of *bla*_*OXA–*__51_ was consistent with their protein production, we took advantage of the monoclonal antibody specific to OXA-51 variants that produced by our lab to examine the production level of these OXA-51 variants by western blotting (Figure 1). It was shown that protein production correlated well with the qRT-PCR data, and that the five strains apparently over-produced the OXA-51 enzymes. The result confirmed that the IS*Aba1* insertion sequence is a key factor that promotes *bla*_*OXA–*__51_ expression. To summarize, although the physiological status of individual strain may affect carbapenem susceptibility to some extent, resistance was observed only among *A. baumannii* strains which carried either IS*Aba1-bla*_*OXA–*__51_ or additional CHDL-encoding genes such as *bla*_*OXA*__23_ and *bla*_*OXA–*__72_.

To confirm that overexpression of OXA-23 and intrinsic OXA-51 is the major mechanisms of carbapenem resistance in clinical *A. baumannii*, a total of 453 carbapenem-resistant clinical *A. baumannii* isolated from four different regions of China were screened for the presence of these two mechanisms (Table 3). Our results showed that 404/453 strains carried over-expressed OXA-23 with IS*Aba1* in the promoter region of *bla*_*OXA–*__23_, while the rest of 49 strains that did not carry *bla*_*OXA–*__23_ exhibited an insertion of IS*Aba1* in the promoter region of *bla*_*OXA–*__51_ and over-expression of OXA-51, which was confirmed by Western-blot using specific antibody to OXA-51 (Table 3). These data suggested that mechanisms of carbapenem resistance in clinical *A. baumannii* were mediated by either over-production of OXA-23 or OXA-51 through insertion of IS*Aba1* in their promoter region.

**TABLE 3:** Surveillance of mechanisms of carbapenem resistance in clinical carbapenem-resistant *A. baumannii* isolates from different regions of China.

**Characteristics of *A. baumannii* isolates from different sources**		**Guangdong, China**	**Zhejiang, China**	**Henan, China**	**Hong Kong, China**	**Total**
Total No of strains of each source		163	173	72	45	453
Average MICs	MER	24.4	18.4	24.8	20.6	21.8
	IMP	26.8	16.2	24.6	22.4	22.0
	ERA	86.4	64.2	78.8	66.4	74.7
No of positive strains	*bla*_*OXA–*__51_	163	173	72	45	453
	IS*Aba1*- *bla*_*OXA–*__51_	18	19	4	8	49
	*bla*_*OXA–*__23_	145	154	68	37	404
	IS*Aba1*- *bla*_*OXA–*__23_	145	154	68	37	404
	Overexpression of OXA-51 (WB)^#^	18	19	4	8	49

*MER, meropenem; IMP, imipenem; ERA, ertapenem. ^#^OXA-51 expression level was determined by Western-blot using OXA-51 specific antibody developed in our lab.*

Correlation analysis of the enzymatic activity of OXA types carbapnemase and meropenem MIC of strains producing these enzymes indicated that: (1) *A. baumannii* strains that over-expressed OXA-23/OXA-24 regardless of the status of over-expression of OXA-51 variants generally displayed higher MIC of meropenem (≥16 μg/ml); (2) *A. baumannii* strains that over-expressed OXA-51 variants only displayed relatively low MIC of meropenem, with OXA-83 (32 μg/ml) being higher than OXA-79/OXA-83 (8 μg/ml); and (3) Western-blot experiments indicated that OXA-51 variants exhibited base line expression without the insertion of IS*Aba1*, which is probably true for extrinsic OXAs. OXA variants with lower activity like OXA51 variants are needed to be overexpressed to counter carbapeneme resistance phenotype, while variants with high catalytic activity such as OXA-72 may not need to be overexpressed, its basic line expression might be enough to mediate high carbapenem resistance in *A. baumannii* in the case of MH2 in this study.

## Conclusion

*Acinetobacter baumannii* is an important pathogen that may cause fatal infections in nosocomial settings. It was thought that the interplay of multiple resistance mechanisms, including over-expression of endogenous efflux systems, suppression of expression of porin proteins, reduced membrane permeability and carriage of CHDL-encoding elements could lead to carbapenem resistance in this bacterial pathogen. In the current study, through systematic characterization of a set of clinical *A. baumannii* strains with different OXA-23 and MIC profiles, we demonstrated that the RND efflux system and membrane porin proteins were not the key factors that conferred carbapenem resistance in the *A. baumannii* strains tested. Instead the OXA-23 and OXA-24 enzymes (OXA-72 variant) were found to be the key elements underlying carbapenem resistance in *A. baumannii* strains as a result of insertion of the IS*Aba1* element, which provides a promoter for over-expression of the *bla*_*OXA–*__23_ and *bla*_*OXA–*__23_ genes (Nigro and Hall, 2016). In *A. baumannii* strains lacking these genes, carbapenem resistance was mainly due to the over-expression of *bla*_*OXA–*__51_ variants, again through insertion of the promoter-bearing IS*Aba1* elements. Findings in this study emphasized the role of *bla*_*OXA*_ type enzymes in mediating carbapenem resistance in this major clinical pathogen. These data provided essential information for the design of new strategies to treat clinical infections caused by carbapenem-resistant *A. baumannii* strains.

## Data Availability Statement

The whole genome shotgun sequences of isolates included in this study have been deposited in GenBank with the following accession numbers: AB1, SAMN09667770; AB2, SAMN09667771; AB3, SAMN09667772; AB4, SAMN09667773; AB5, SAMN09667774; AB7, SAMN09667775; AB8, SAMN-09667776; AB10, SAMN09667777; MH1, SAMN09667778; MH2, SAMN09667779; MH3, SAMN09667780; MH5, SAMN09667781; MH6, SAMN09667782; and MH7, SAMN09667783.

## Author Contributions

MW and BC designed and performed the research, and drafted the manuscript. EC edited the manuscript. SC supervised the study and edited the manuscript.

## Conflict of Interest

The authors declare that the research was conducted in the absence of any commercial or financial relationships that could be construed as a potential conflict of interest.
