# Supplementary material for: Over-Expression of ISAba1-Linked Intrinsic and Exogenously Acquired OXA Type Carbapenem-Hydrolyzing-Class D-ß-Lactamase-Encoding Genes Is Key Mechanism Underlying Carbapenem Resistance in Acinetobacter baumannii
Source: Front Microbiol. 2019 Dec 4;10:2809. doi: 10.3389/fmicb.2019.02809 (PMC6904305; doi:10.3389/fmicb.2019.02809)
Supplement: Supplementary file 1 [file Data_Sheet_1.pdf]

Supplementary materials

Over-expression of IS*Aba1*-linked intrinsic and exogenously acquired OXA type Carbapenem-Hydrolysing-Class D-ß-Lactamase-encoding genes is key mechanism underlying carbapenem resistance in *Acinetobacter baumannii*

Marcus Ho-yin WONG[1#], Bill Kwan-wai CHAN[1#], Edward Wai-chi CHAN[1], Sheng CHEN[1,2*]

Running Title: CHDL-mediated carbapenem resistance

[1]State Key Laboratory of Chemical Biology and Drug Discovery, The Hong Kong Polytechnic University, Hung Hom, Kowloon, Hong Kong.

2 Department of Infectious Diseases and Public Health, Jockey Club College of Veterinary Medicine and Life Sciences, City University of Hong Kong, Kowloon, Hong Kong

#contribute equally to the work.

**Corresponding author: Sheng CHEN, Tel: (852)-3442-5782; Email: shechen@cityu.edu.hk

Keywords: *A. baumannii*, carbapenem resistance, OXA-23, OXA-51, mechanisms

Supplementary Table S1. Primers used in this study.

Primer	Sequence 5'to 3	Applications
IS*Aba1*-F	CACGAATGCAGAAGTTG	PCR screening
OXA51-F	CTCACCTTATATAGTGACTGCTAATCC	PCR screening
OXA51-R	CTATAAAATACCTAATTGTTC	PCR screening
OXA23-F	CTTGCTATGTGGTTGCTTCT	PCR screening
OXA23-R	ATTTCTGACCGCATTTCCAT	PCR screening
Oxa-23F	TAATGCTCTAAGCCGCGCAA	qRT-PCR
Oxa-23R	TGACCTTTTCTCGCCCTTCC	qRT-PCR
Oxa-51like F	TTTTGGCTGGTGGGTCCTTT	qRT-PCR
Oxa-51like R	CATCCCATCCCCAACCACTT	qRT-PCR
carO-F	CTGCAATGGCGGATGAAGC	qRT-PCR
carO-R	GCCATAACAAAGCACCACCG	qRT-PCR
AdeR-F	CGAGCCAACCCATCGATTTA	qRT-PCR
adeR-R	GGCTTCACCACAAAGTCATC	qRT-PCR
AdeB-F	GGATTATGGCGACAGAAGGA	qRT-PCR
AdeB-R	AATACTGCCGCCAATACCAG	qRT-PCR
GyrB-F	GATGATGCGCGTGAAGGTTT	qRT-PCR
GyrB-R	CATTGCTTGCTCTACCGCTG	qRT-PCR

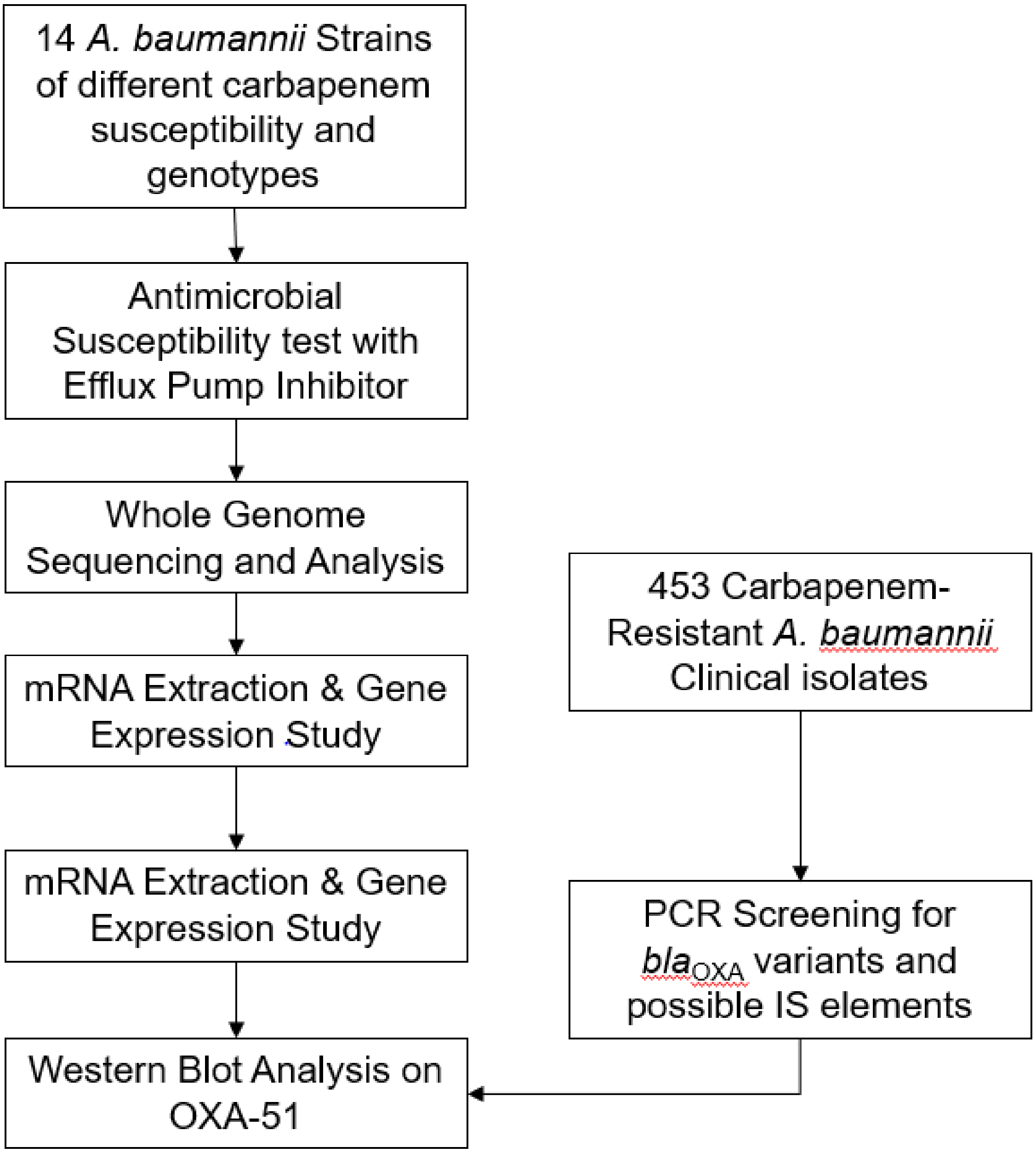

Supplementary Figure S1. Flowchart of methodology illustrating the workflow of the study.

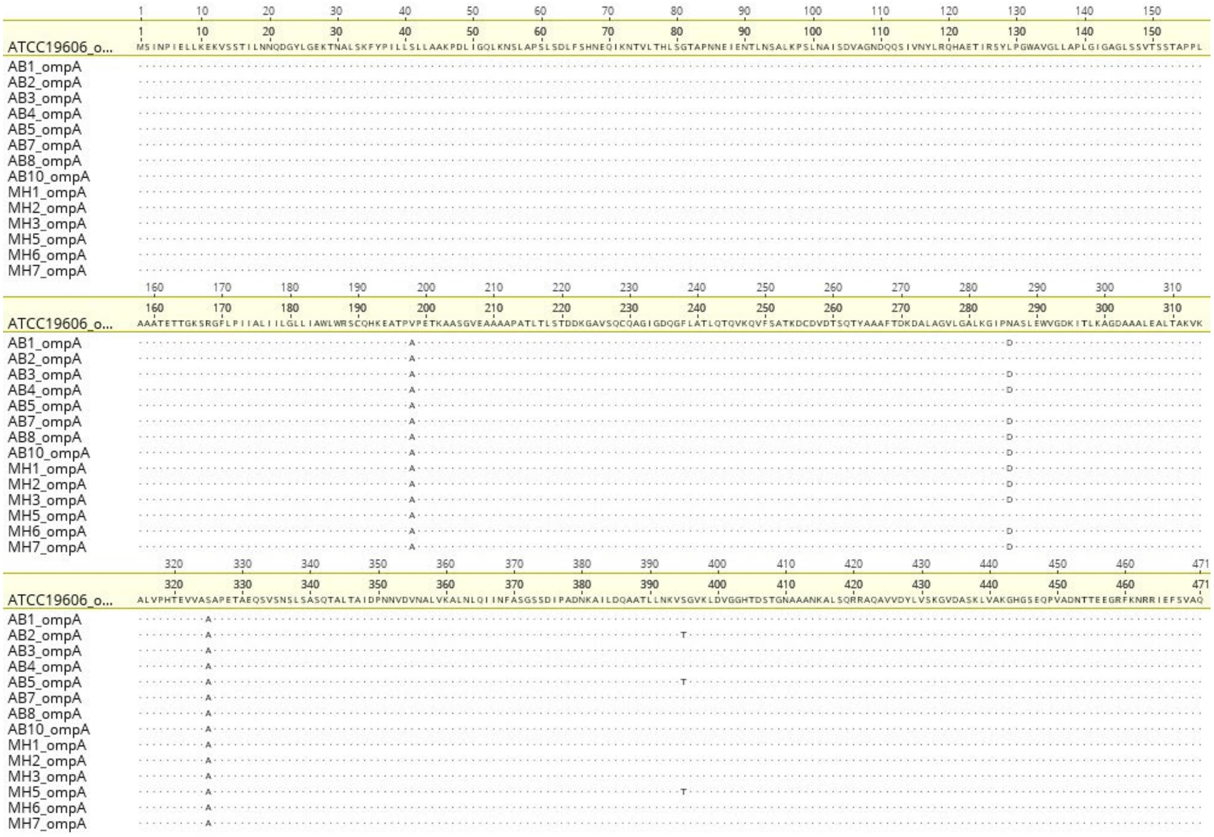

Supplementary Figure S2. Amino acid Sequence alignment of OmpA of *A. baumannii* strains tested in this study.

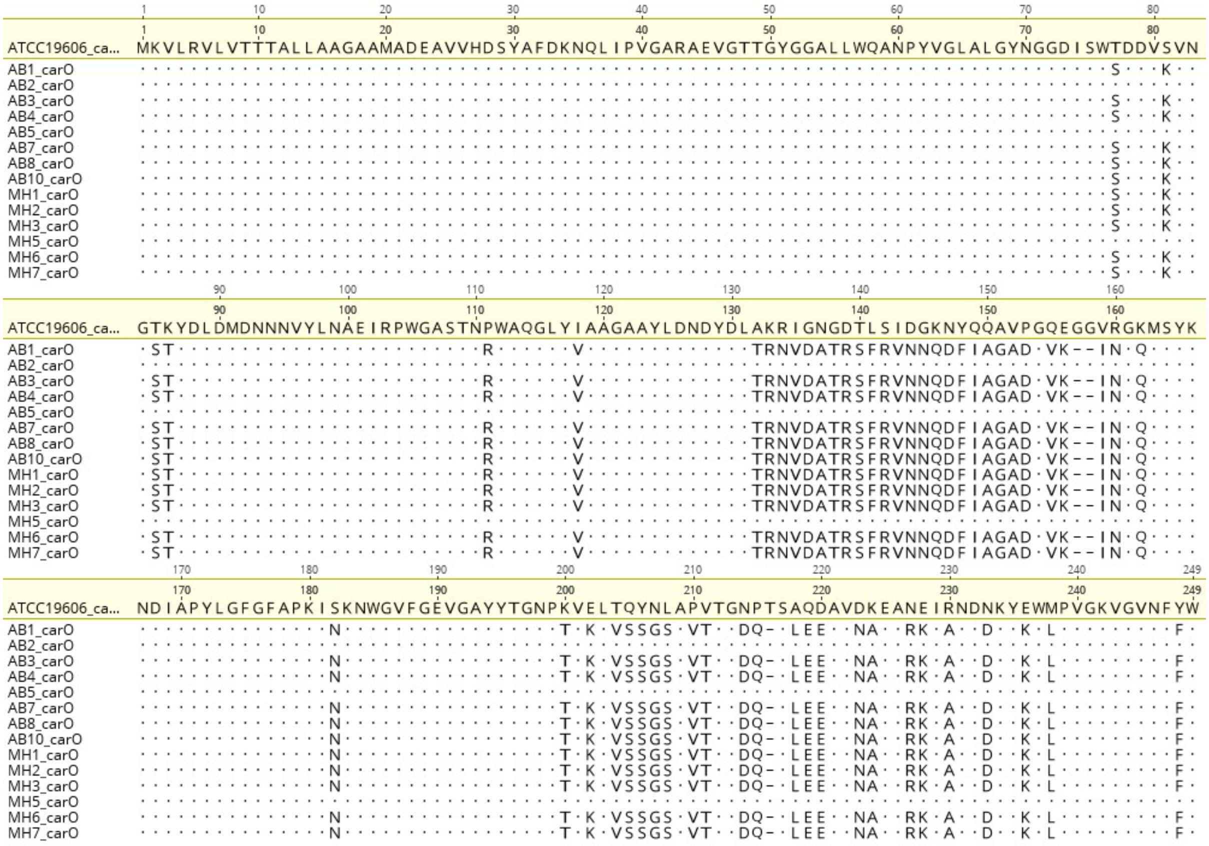

Supplementary Figure S3. Amino acid Sequence alignment of CarO of *A. baumannii* strains tested in this study.

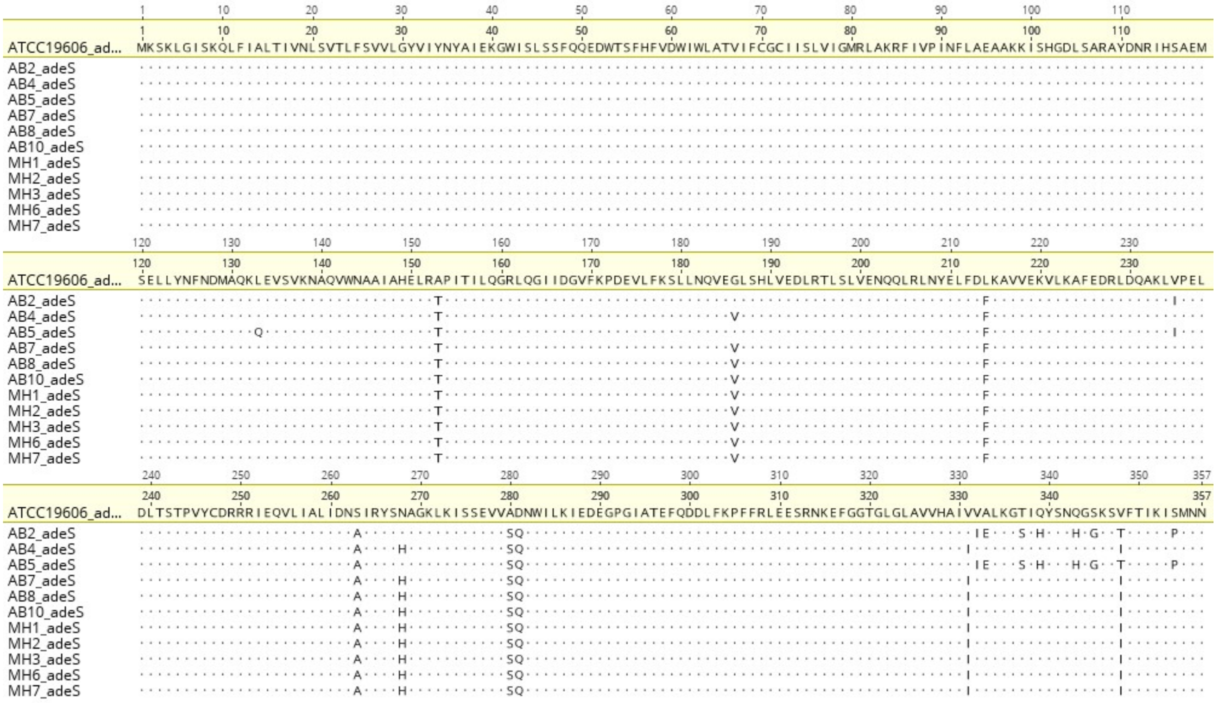

Supplementary Figure S4. Amino acid Sequence alignment of AdeS of *A. baumannii* strains tested in this study.

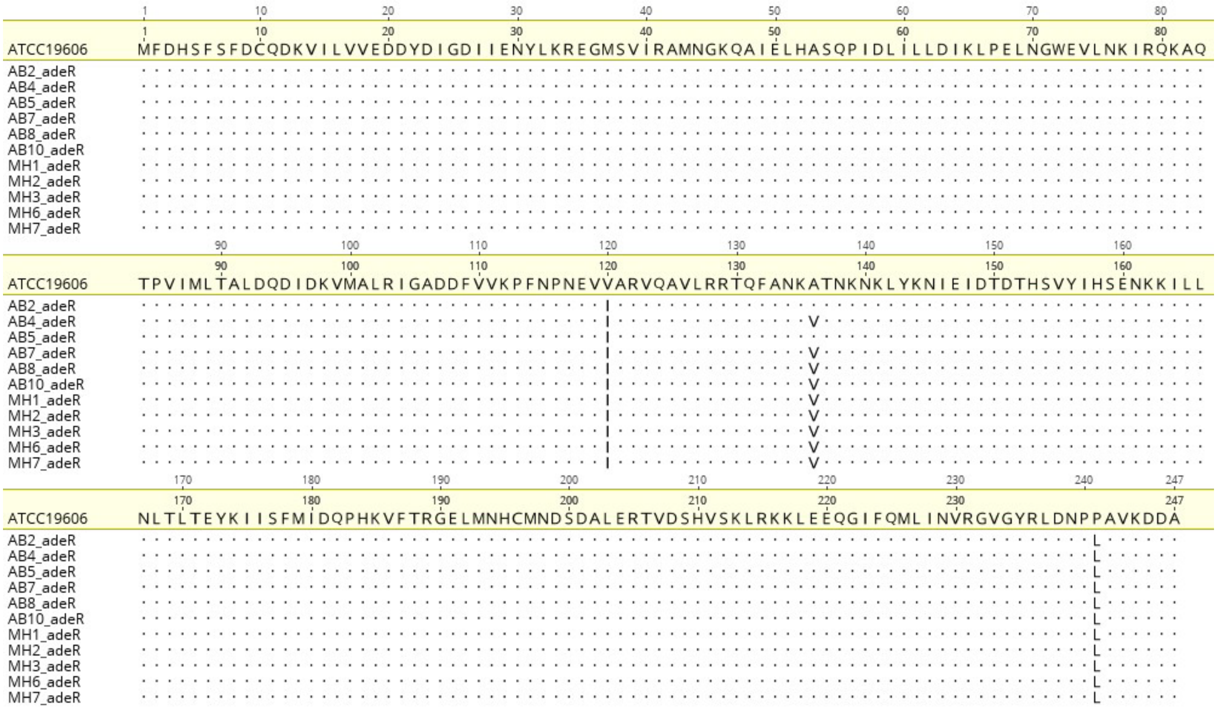

Supplementary Figure S5. Amino acid Sequence alignment of AdeR of *A. baumannii* strains tested in this study.

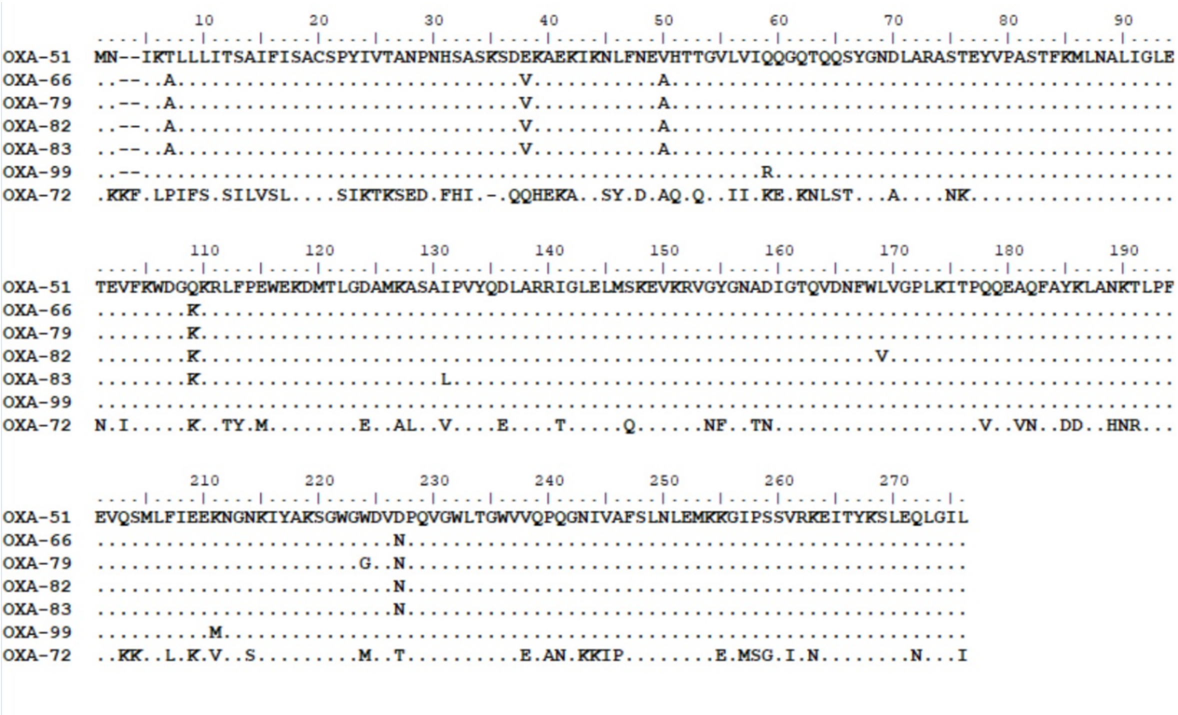

Supplementary Figure S6. Amino sequence alignment of OXA-51 identified in this study. Alignment was performed by Cluster W. Sequence of OXA-51 is included as reference sequence.

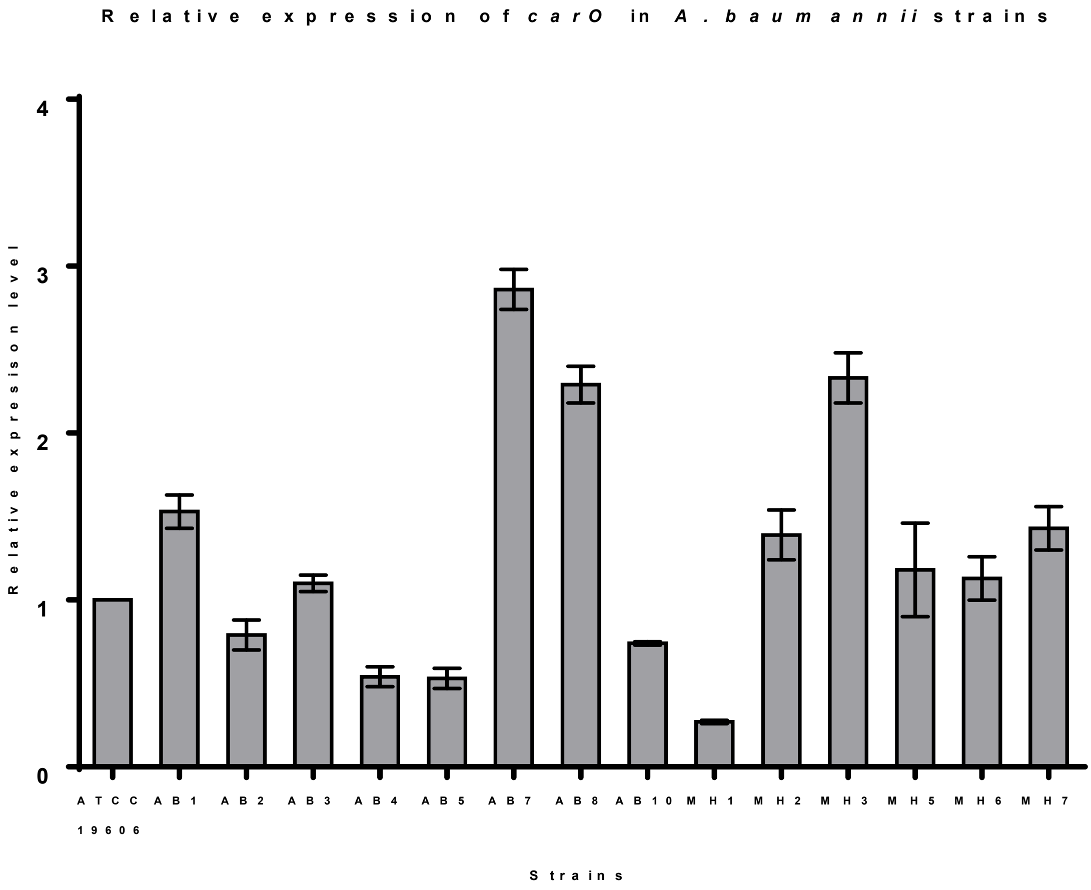

Supplementary Figure S7. Relative expression of *carO* in *A. baumannii* strains. The qRT-PCR was performed by Roche LightCycler 480 system.

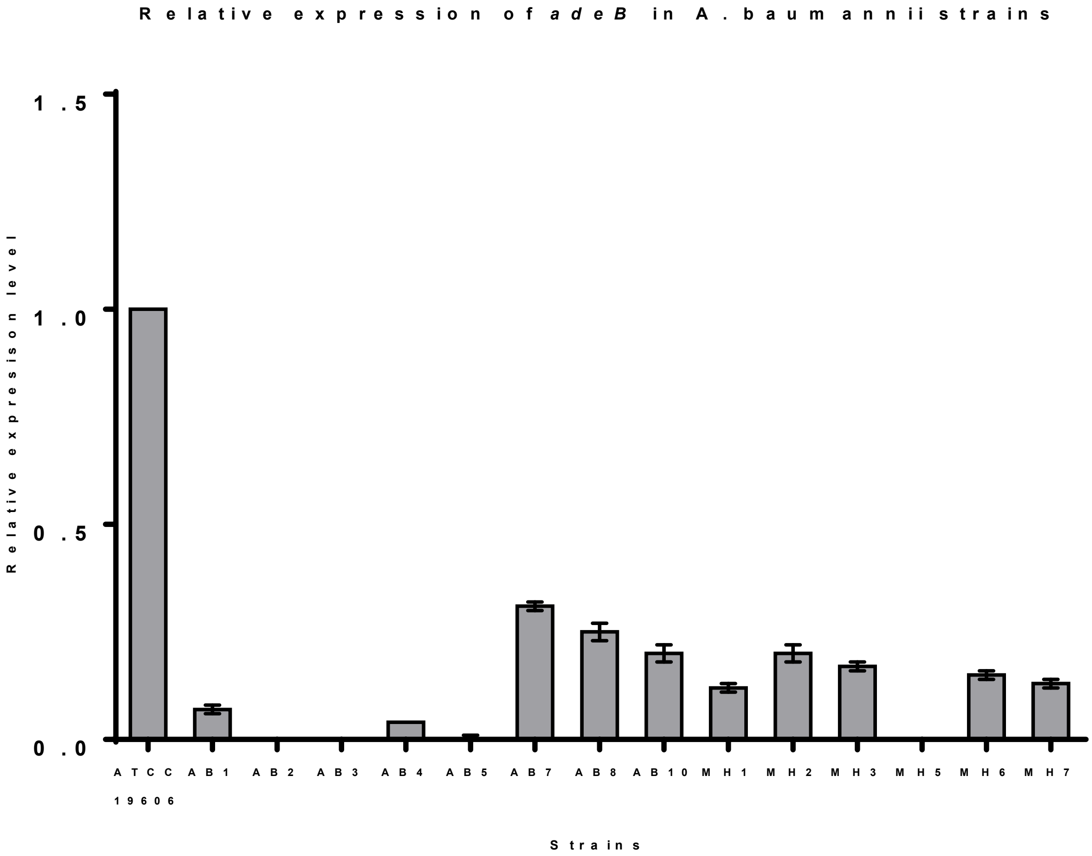

Supplementary Figure S8. Relative expression of *adeB* in *A. baumannii* strains. The qRT-PCR was performed by Roche LightCycler 480 system.

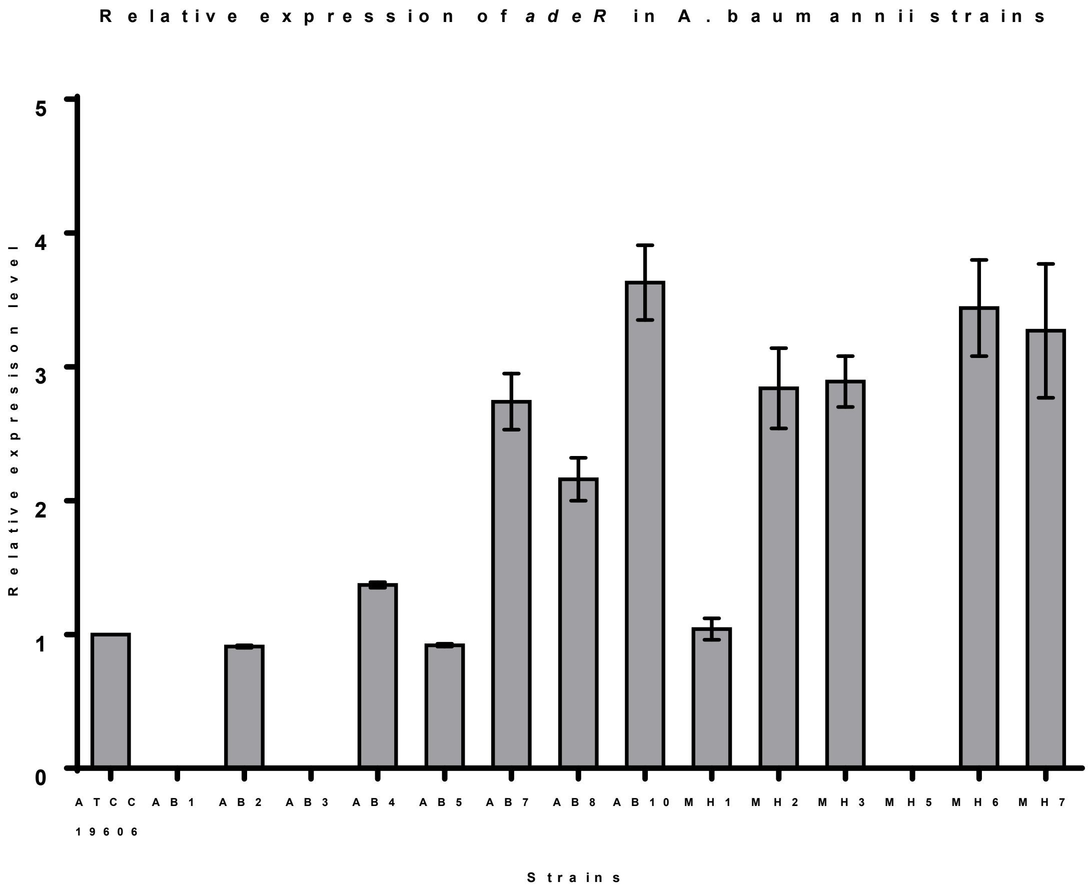

Supplementary Figure S9. Relative expression of *adeR* in *A. baumannii* strains. The qRT-PCR was performed by Roche LightCycler 480 system.

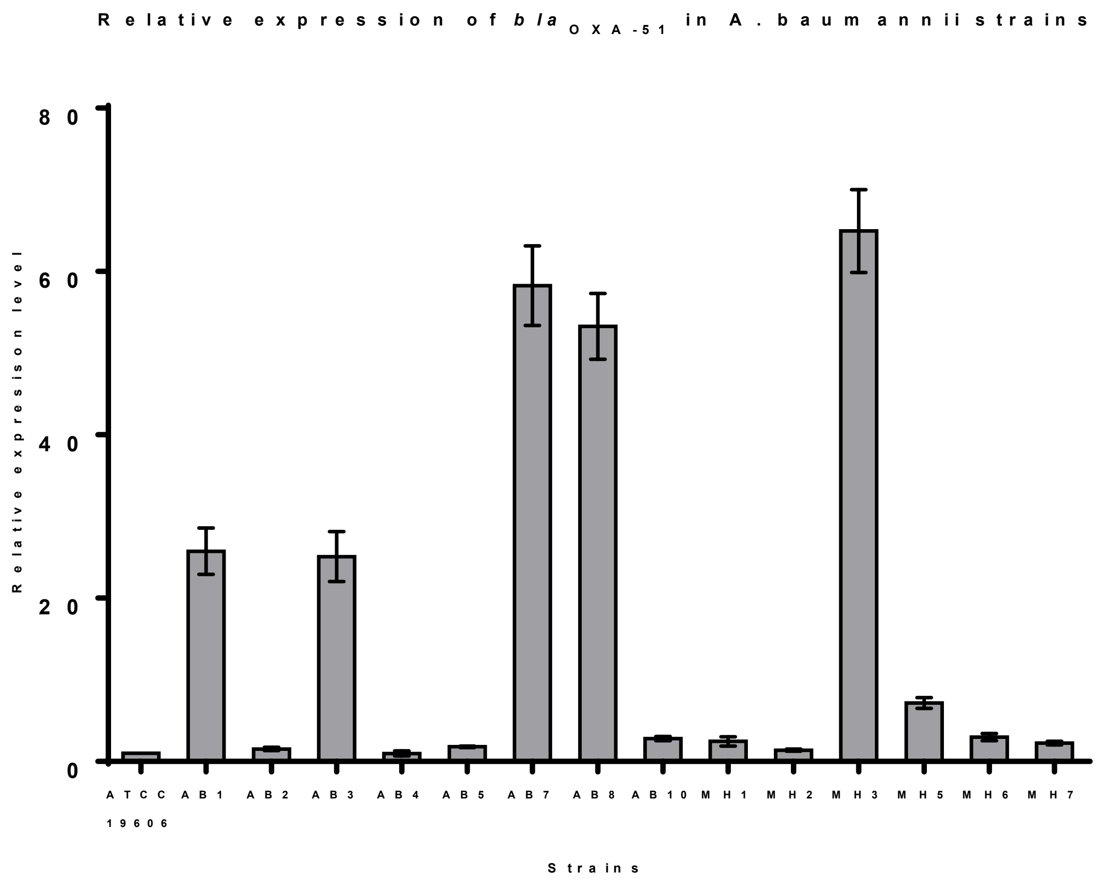

Supplementary Figure S10. Relative expression of *bla*$_{OXA-51}$ in *A. baumannii* strains. The qRT-PCR was performed by Roche LightCycler 480 system.

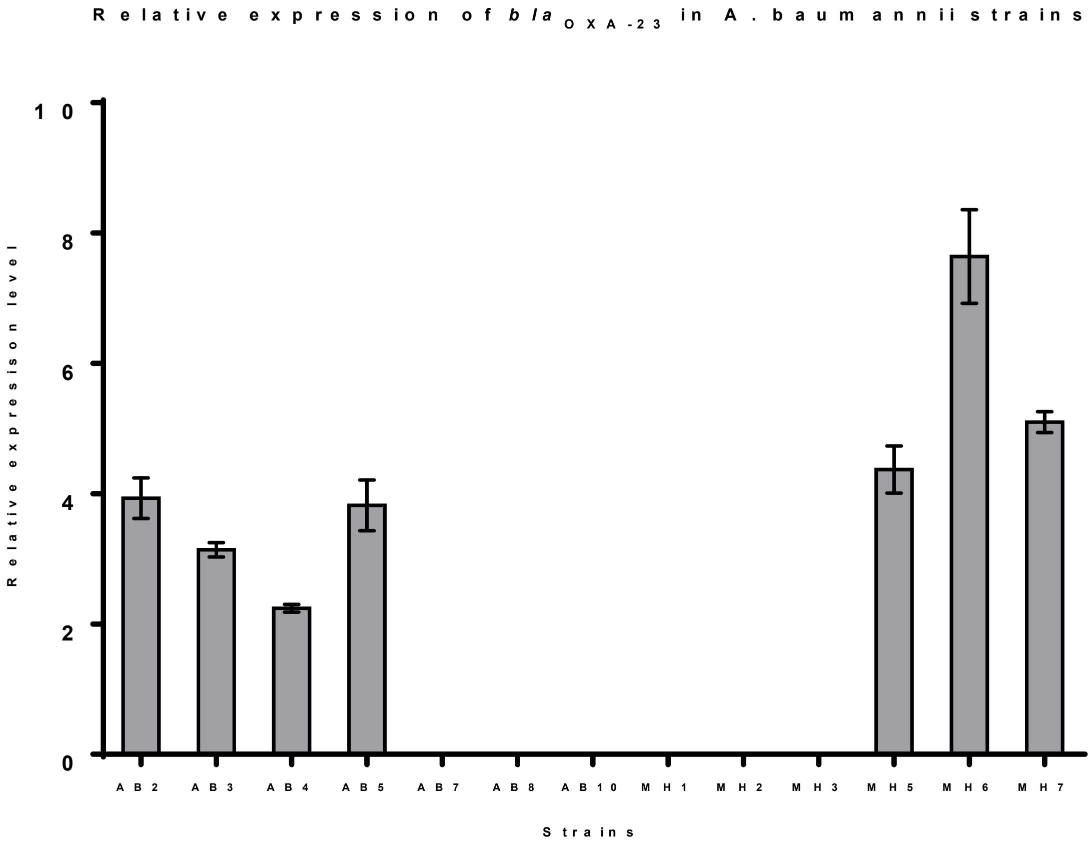

Supplementary Figure S11. Relative expression of *bla*$_{OXA-23}$ in *A. baumannii* strains. The qRT-PCR was performed by Roche LightCycler 480 system.
